# A novel method of non-clinical dispatch is associated with a higher rate of critical Helicopter Emergency Medical Service intervention

**DOI:** 10.1186/s13049-018-0551-9

**Published:** 2018-09-25

**Authors:** Scott Munro, Mark Joy, Richard de Coverly, Mark Salmon, Julia Williams, Richard M. Lyon

**Affiliations:** 1 School of Health Sciences, Faculty of Health and Medical Sciences, University of Surrey, Guildford, Surrey GU2 7XH UK; 2 Kent, Surrey & Sussex Air Ambulance Trust, Redhill Airfield, Redhill, Surrey RH1 5YP UK; 3 South East Coast Ambulance Service NHS Foundation Trust, Banstead, Surrey SM7 2AS UK; 4 School of Health and Social Work, University of Hertfordshire, Hatfield, Hertfordshire AL10 9AB England

**Keywords:** Air ambulance, Helicopter emergency medical services, Emergency medical services, Dispatch, Tasking

## Abstract

**Background:**

Helicopter Emergency Medical Services (HEMS) are a scarce resource that can provide advanced emergency medical care to unwell or injured patients. Accurate tasking of HEMS is required to incidents where advanced pre-hospital clinical care is needed. We sought to evaluate any association between non-clinically trained dispatchers, following a bespoke algorithm, compared with HEMS paramedic dispatchers with respect to incidents requiring a critical HEMS intervention.

**Methods:**

Retrospective analysis of prospectively collected data from two 12-month periods was performed (Period one: 1st April 2014 – 1st April 2015; Period two: 1st April 2016 – 1st April 2017). Period 1 was a Paramedic-led dispatch process. Period 2 was a non-clinical HEMS dispatcher assisted by a bespoke algorithm. Kent, Surrey & Sussex HEMS (KSS HEMS) is tasked to approximately 2500 cases annually and operates 24/7 across south-east England. The primary outcome measure was incidence of a HEMS intervention.

**Results:**

A total of 4703 incidents were included; 2510 in period one and 2184 in period two. Variation in tasking was reduced by introducing non-clinical dispatchers. There was no difference in median time from 999 call to HEMS activation between period one and two (period one; median 7 min (IQR 4–17) vs period two; median 7 min (IQR 4–18). Non-clinical dispatch improved accuracy of HEMS tasking to a mission where a critical care intervention was required (OR 1.25, 95% CI 1.04–1.51, *p* = 0.02).

**Conclusion:**

The introduction of non-clinical, HEMS-specific dispatch, aided by a bespoke algorithm improved accuracy of HEMS tasking. Further research is warranted to explore where this model could be effective in other HEMS services.

## Background

Major trauma is a leading cause of mortality and serious morbidity, especially in the young [1]. Emergency Medical Services can influence the outcome of major trauma patients by the type and quality of pre-hospital care they deliver [2–4]. In many regions, advanced medical teams, often including a doctor, respond to accidents as part of a Helicopter Emergency Medical Service (HEMS) [5]. For trauma victims and patients who are medically critically unwell, HEMS can deliver specialist clinical care, such as pre-hospital anaesthesia, blood product transfusion and advanced clinical intervention, as well as the ability to rapidly transport the patient to hospital [6].

The role of deploying a HEMS team is a complex and nuanced task. As HEMS is a scarce resource, it is important that they are only tasked to missions with a high likelihood of requiring advanced clinical intervention, beyond the scope of standard land ambulance crews. This also has important consequences for the accurate triage of major trauma patients [7]. Within the air ambulances in the United Kingdom (UK) there is a wide variation in the criteria used for tasking, crew configurations and dispatch arrangements [8]. Different dispatch personnel and criteria have been previously studied, however as there is no standardised tasking approach across the different UK HEMS providers, it is difficult to compare results [9].

In order to make optimum use of a scarce and costly clinical resource, the criteria for dispatching HEMS to trauma scenes should have a high sensitivity and specificity in order to reduce over-triage; which may result in high costs and increased risk to crew safety, and under-triage; which may result in patients not receiving the assistance of a specialist HEMS team when needed [10].

A common HEMS dispatch model is to have a dedicated person situated within an ambulance service emergency operation centre (EOC) to screen incoming emergency calls and assess them for suitability of requiring a HEMS response [11]. This model is currently used across many UK and international HEMS services [8, 10]. A “HEMS desk” is staffed by a HEMS-trained paramedic who screens incoming emergency calls from the ambulance service computer-aided dispatch system (CAD).

There are limitations of this HEMS dispatch model. There is a lack of a standardisation to HEMS dispatch, with individual clinicians making their own judgment on the need to task HEMS. This system is open to selection bias and significant inter-operator variability [8].

Two recent systematic reviews concluded that due to a paucity of high quality research in this field, it is not possible to identify a model that best optimises resource utilisation, with currently no validated criteria to guide the development of definitive guidelines [11].

Due to the limited evidence surrounding optimal HEMS dispatch models, this study aimed to investigate any association of clinical training and experience of dispatchers on accurately dispatching the HEMS team to incidents where HEMS-specific interventions were required. We sought to evaluate whether, in a UK HEMS service, non-clinically trained dispatchers using a bespoke HEMS tasking algorithm, compared with HEMS paramedic dispatchers, were as effective at accurately dispatching HEMS to missions requiring critical intervention.

## Methods

### Setting

Kent, Surrey & Sussex Air Ambulance Trust (KSSAAT) provides two doctor-paramedic teams, one of which operates 24-h a day and the other 12-h a day, responding to emergency calls across southeast England. KSSAAT responds to 70% trauma and 20% medical missions. Missions are selected by a dedicated KSSAAT dispatcher who is present in the South East Coast Ambulance Service (SECAmb) control room and continuously screens incoming emergency calls. The KSSAAT teams can deploy by helicopter or response car. The team consists of a pilot, a physician from an emergency medicine or anaesthesia background and a paramedic who has undergone additional service-specific training. The crews bring advanced clinical procedures directly to patients, such as pre-hospital anaesthesia, advanced analgesia, advanced airway management, blood transfusion and surgical interventions. The KSSAAT HEMS service provides enhanced pre-hospital medical care to south east England, a static population of approximately 4.3 million and a transient population of up to a total of 10 million. Patient transport to hospital can be by air or road, depending on geography, weather, time of day and hospital helipad availability. Statistics from the UK National Audit Office suggest that in this region of the UK, there are approximately 630 cases of major trauma annually.

### HEMS dispatch model

KSSAAT previously used HEMS paramedic dispatchers (HPD) working on a dedicated dispatch desk in the EOC of the local ambulance service to activate the helicopter and its crew. Since January 2016, due to rota constraints and the availability of HEMS paramedics, it was no longer possible to fully cover the HEMS dispatch desk with a designated HEMS paramedic. Other means of specialist dispatch were therefore explored and the trust began training non-clinically trained dispatchers (NCDs) to work on the HEMS dispatch desk. All NCDs came from an ambulance dispatch background, with all candidates having extensive experience of working an ambulance control room. This study compares a total of 20 individual HPDs to 5 NCDs.

**Fig. 1:**
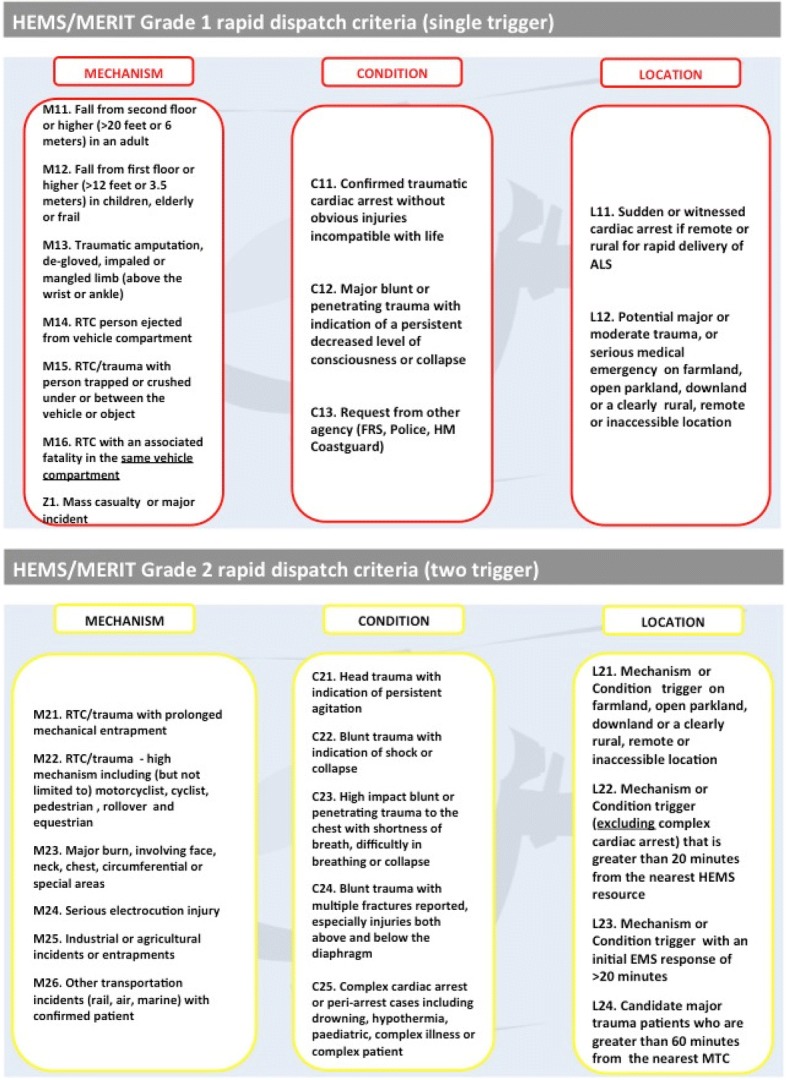
KSSAAT HEMS tasking criteria used by non-clinical HEMS dispatchers. Grade 1 dispatch requires a single trigger to be met. Grade 2 dispatch requires two triggers to be met

At the time of the study, all the NCDs came from a background working in the ambulance service EOC, dispatching land ambulances. As part of their HEMS dispatch training they were put through an induction course, followed by a four-week development period, starting with observation of the dispatch desk progressing to peer supervised practice and culminating in a sign-off assessment undertaken by an operational manager. The NCDs were aided by a bespoke tasking algorithm, devised by the KSSAAT team. The algorithm was based on expert opinion and internal consensus. This algorithm classifies HEMS dispatch into Grade 1 and Grade 2 dispatches for HEMS, based on mechanism of injury, clinical condition of the patient and geographical location. The specifics of the HEMS tasking criteria are shown in Fig. 1. The algorithm is paper based. Whilst listening to the incoming emergency call, dispatchers aim to rapidly identify either one (from Grade 1 criteria list) or two (from Grade 2 criteria list). If these are positively identified, HEMS is dispatched. Grade 1 should be dispatched within 5 min and Grade 2 within 10 min of receipts of 112/999 call.

In addition, NCDs were fully integrated into the KSSAAT Clinical Governance system. This includes receiving feedback on individual missions, attending Clinical Governance days (CGD) and receiving on-going training. CGDs are held every 2 weeks with dispatchers attending to partake in clinical case reviews. On average, 10–12 missions are reviewed on each CGD. Dispatchers attend a 1-h update/training session as part of each CGD and share experience with the other dispatchers. Peer review of dispatchers occurs 1–2 times per month by having a member of the KSSAAT senior management team undertake a dispatch shift with them.

In order to investigate any association between type of dispatcher and accurate dispatch, a service evaluation of incidents attended by the KSSAAT was undertaken as a prospectively planned project.

### Data capture

A retrospective analysis of collected data from two 12-month periods was performed (Period one: 1st April 2014 – 1st April 2015; Period two: 1st April 2016 – 1st April 2017). Period one consisted of data where clinically trained HPDs were responsible for dispatching the HEMS crew, period two was collected from when NCD were responsible. Data were collected from KSSAAT electronic records, which are captured, on a custom-built electronic database (HEMSbase, MedicOne Systems Ltd., UK). KSSAAT dispatchers and clinical teams all use this web-based database which has pre-assigned fields and drop down menus to allow standardised data entry. The dispatcher enters call details and dispatch details and the HEMS team complete the clinical sections. There were no changes to the data capture process during the study period. All jobs attended by KSSAAT during these two periods were included in the analysis.

### Outcome measures

The primary outcome for this study was accurate dispatch of the HEMS team to incidents that required pre-specified, pre-hospital interventions that would only be available to the patients in this region by a HEMS team. These included any of pre-hospital anaesthesia; the administration of blood products; resuscitative thoracotomy; chest thoracostomy and a ‘code red’ alert to a major trauma centre. If a single intervention was performed, a positive primary outcome was recorded. The secondary outcome was the time from initial 999/112 call to activation of the HEMS team.

### Data analysis

Dispatch data are presented using descriptive statistics, a chi-square test was used to compare categorical variables between the HPD and NCD groups and a Kruskal-Wallis test for continuous variables. The threshold for statistical significance was set at 5%.

A multiple logistic regression was used to analyse the relationship between accurate dispatch to incidents requiring HEMS intervention and dispatcher type. The dependent variable was accurate dispatch (accurate/ not accurate) and the independent variables were dispatcher type (NCD/HPD); job type (Assault/ exposure/ intentional self-harm/ medical/ other/ other transport/ road traffic collision/ sport or leisure); categorisation of dispatch and result of incident (patient conveyed to hospital/patient treated on scene/stood down en route/ stood down at scene). Odds ratios (OR) with 95% confidence intervals (CI) are reported. The statistical software package ‘R’ (R Core Team, 2016, version 3.4.2) was used to undertake all statistical analysis.

## Results

A total of 4703 incidents were included in this study; 2519 in period one and 2184 in period two. Period one consisted of 335 more incidents being tasked than in period two. The overall summary of HEMS dispatches is shown in Table 1. The introduction of non-clinical dispatch was associated with a higher proportion of Category 1 dispatches (OR 1.74, 95% CI 1.54–1.97), a lower proportion of Category 2 dispatches (OR 0.49, 95% CI 0.44–0.55) and a rise in the number of land ambulance crews requesting HEMS (OR 1.29, 95% CI 1.13–1.48).

**Table 1:** Summary of HEMS dispatch comparing HEMS paramedic dispatch vs non-clinical dispatch

	Total (*n* = 4703)	HEMS paramedic dispatch (*n* = 2519)	Non-clinical dispatch (*n* = 2184)	*p*-value
Result				
Patient conveyed	1681 (36%)	960 (38%)	721 (33%)	NS
Patient Treated	1228 (26%)	635 (25%)	593 (27%)	NS
Stand down at scene	113 (2%)	68 (3%)	45 (2%)	NS
Stand down en route	1681 (36%)	856 (34%)	825 (38%)	< 0.001
Interventions				
RSI	490 (10%)	245 (10%)	245 (11%)	NS
Blood products	169 (4%)	77 (3%)	92 (4%)	NS
Code red	122 (3%)	45 (2%)	77 (4%)	NS
Thoracotomy	4 (0.1%)	4 (0.2%)	0 (0%)	NS
Thoracostomy	262 (6%)	116 (5%)	146 (7%)	0.01
Job type				
RTC	1988 (42%)	1060 (42%)	928 (42%)	NS
Accidental injury	1158 (25%)	589 (23%)	569 (26%)	NS
Medical	522 (11%)	285 (11%)	237 (11%)	NS
Assault	387 (8%)	192 (8%)	195 (9%)	NS
Intentional self-harm	258 (5%)	155 (6%)	103 (5%)	NS
Sport/leisure	257 (5%)	151 (6%)	106 (5%)	NS
Other	106 (2%)	70 (3%)	36 (2%)	NS
Exposure	27 (0.6%)	17 (1%)	10 (0.5%)	0.003
Job dispatch				
Cat 1 (immediate)	1586 (34%)	704 (30%)	882 (40%)	< 0.001
Cat 2 (interrogate)	2056 (44%)	1300 (52%)	756 (35%)	< 0.001
Cat 3 (crew request)	1061 (23%)	515 (20%)	546 (25%)	< 0.001
Time intervals				
999 to HEMS, median, IQR	7 (4–17)	7 (4–17)	7 (4–18)	0.36
999 to scene, median, IQR	36 (26–51)	36 (26–51)	36 (26–52)	0.97

*HEMS* helicopter emergency medical service*, RSI* rapid sequence induction of anaesthesia*, IQR* interquartile range

The frequency of the HEMS team conveying patients to hospital was higher during period one group. The HEMS team were stood down en route to incidents more frequently in period two.

A multi-variant logistic regressions showed that there was an association between NCD and accurate dispatch to an incident where a HEMS intervention (defined as the patient needing RSI, blood transfusion, thoracostomy or resuscitative thoracotomy) was required (OR 1.25, 95% CI 1.04–1.51, *p* = 0.02). The unadjusted figures for correct dispatch also favoured NCD and are are shown in Table 2.

**Table 2:** Unadjusted comparison of clinical vs non-clinical dispatcher for accurate HEMS dispatch

Unadjusted correct dispatch for HEMS clinical intervention		
	Yes	No
Clinical	322	2197
Non-clinical	332	1852

Chi-square = 5.71, *p* = 0.017

There was no difference in median time from 999 call to HEMS activation between period one and two (period one; median 7 min (IQR 4–17) vs period two; median 7 min (IQR 4–18). This is shown in Table 3.

**Table 3:** Time from initial emergency 999 call to HEMS activation

	Overall	HPD	NCP	*p*-value
Minutes from 999 to HEMS activation, median, IQR	7 (4–17)	7 (4–17)	7 (4–18)	0.3
Minutes from 999 to HEMS on scene, median, IQR	36 (26–51)	36 (26–51)	36 (26–52)	1

*HEMS* helicopter emergency medical service*, HPD* HEMS paramedic dispatcher*, NCP* non-clinically trained dispatcher*, IQR* interquartile range

## Discussion

The results of this study suggest that non-clinically trained dispatchers, assisted by a bespoke HEMS tasking algorithm and fully integrated into a HEMS service, are more effective at accurately dispatching a HEMS team, as a HEMS-trained paramedic, working in the EOC. This is a significant finding as the wider use of this system could provide a more consistent and cost-effective approach to HEMS tasking.

Accurate triage is important for any trauma system [7, 12]. Over triage can lead to unnecessary burden on specialist major trauma centres and is inconvenient for patients and their families. Under triage can lead to significant clinical risk for patients if they require specialist intervention, which is not available at the receiving hospital [7, 12]. HEMS services are thought to triage more accurately than land-bases services [12]. In KSS, we have a performance indicator aiming to keep over triage < 15% and under triage < 5%.

Previous studies have shown outcome benefit when HEMS is tasked to the correct missions [13]. Andruszkow et al. (2014) undertook a large retrospective analysis of a trauma database in Germany to investigate the changes in HEMS-associated pre-hospital trauma care over the last decade and whether a physician-staffed HEMS system has a beneficial impact on outcome in multiple traumatised patients. The results demonstrate an independent survival benefit of HEMS after multiple trauma. In order to maximise the value of HEMS, they need to be tasked to the correct patients who will benefit from the interventions that HEMS offer.

Accurate dispatch is the first step to optimising pre-hospital triage of major trauma patients. HEMS dispatch is known to be problematic [14]. The acceptance of inappropriate activation is system-specific [15]. Where an incident is geographically remote, we prefer an early activation, with stand down en route if further clinical information from a land ambulance crew indicates HEMS is not required, as this saves on time.

Having a HPD has been reported to be the most common model used by HEMS teams across the UK [8], however these results suggest that it may be possible to recruit and train dispatchers, with no prior clinical experience or training, to accurately dispatch HEMS to incidents where HEMS-specific interventions are required. Individual HEMS paramedics rely on their clinical experience and apply this to the tasking process in order to select missions, which may be suitable for HEMS. The use of a bespoke HEMS tasking algorithm is likely to have supported the NCD process. Clinical dispatchers may benefit from such an algorithm and further research is warranted to explore the effect of this algorithm. The drop of individuals from 20 HPD to 5 NCD allowed for a more concentrated experience in HEMS tasking.

It is important to remember that NCD in this study were not ordinary ambulance dispatchers, as analysed in previous studies [16]. NCDs within the KSSAAT service are fully employed by KSSAAT and fall under the KSSAAT Clinical Governance structure, as we have described. They attend Clinical Governance days on a monthly basis, receive direct feedback on their HEMS dispatch performance and undergo regular update training. NCDs are solely responsible for HEMS dispatching and have no responsibility for land ambulance crew tasking. As such, this has allowed a small group of NCDs to become expert in HEMS tasking.

We observed a rise in Category 1 dispatches; suggesting NCDs are more willing or able to accurately and rapidly follow prescribed immediate HEMS tasking criteria. Clinical HEMS dispatchers appear more likely to wait until a land ambulance crew arrives on-scene in order to interrogate the call further, by gaining more clinical information from scene to inform the tasking decision. We believe this can lead to significant variation in interpretation. As the HPD also forms part of the HEMS crew rota, there may be unconscious bias to task HEMS, especially during times of low activity.

We observed a rise in the number of HEMS activations as a result of land ambulance crew requests in Period 2. Unlike HPDs, NCDs lack the clinical ability to discuss a specific case with the requesting land ambulance crew and give clinical advice. This resulted in land ambulance crew requests being activated upon more readily. There is a need for further research to explore the optimum means of extracting the relevant clinical information from a 999/112 caller.

The limitations of this study include its retrospective and observational design. As this study was designed as a service evaluation, the external validity of the results need to be interpreted with caution and further research is warranted to further explore the clinical implications of this study. We accept that using HEMS interventions as the sole proxy marker of a positive dispatch is a limitation. In future, patient outcome would be the desired primary outcome measure, however this was outside the possibility of this initial study. We appreciate the defined HEMS interventions are a simplistic measure of assessing accuracy. However, these criteria are unequivocally associated with the need for HEMS and were therefore chosen. We accept that the decision on clinical intervention is at the discretion of the attending HEMS team. However, our system operates within clear and defined Standard Operating Procedures so we feel the effect of on-scene decision-making is likely to be similar and minimal across both groups.

HEMS clearly bring more to a scene than just specific clinical interventions. Advanced clinical decision making, up- and down-triage, supporting road ambulance crews and coordinating multi-patient incidents are additional benefits of HEMS. Assessing these elements objectively is challenging. We have recently introduced pre-hospital video recording to assist with research in this area in future.

Another limitation is the inability to assess the incidents where a HEMS team would have been of benefit to the patient, but were missed by the dispatchers. We did not have access to ambulance service data to cover critically unwell or injured patients who did not receive HEMS assistance. This prevented detailed sensitivity and specificity calculations. Future studies could use hospital data from trauma data registries to link with pre-hospital data in order to identify patients who were brought to hospital by other means of transportation, but would have otherwise benefited from having a HEMS team present. There are also clearly other benefits that a HEMS team brings to a major trauma scene. These include clinical decision-making, triage and the ability to rapidly transport a patient to hospital by air. It is well recognised that short pre-hospital times can improve outcome, not just for patients with major trauma [17], but also for other time-critical medical conditions such as stroke and acute coronary syndromes.

While there has been a focus on investigating the validity of different criteria for dispatching, such as mechanism of injury, call interrogation and crew request, this study has provided data regarding the training and experience of the person doing the dispatching which in itself may influence dispatchers’ decision making.

A strength of the KSS HEMS system is that all HEMS teams operate under Standard Operating Procedures, meaning interventions are very comparable. There were no significant changes in the HEMS system between period 1 and 2, other than dispatch, allowing us to be more confident of a valid result.

## Conclusion

The introduction of non-clinical, HEMS-specific dispatch, aided by a bespoke algorithm and Clinical Governance system improved the accuracy of HEMS tasking. This model has the potential to be evaluated in other HEMS services and could significantly improve the accuracy of tasking a valuable, pre-hospital resource to the most seriously unwell or injured patients. Further research is warranted to explore where this model could be effective in other HEMS services.

## Abbreviations

CAD: Computer Aided Dispatch
CI: Confidence Interval
EOC: Emergency Operations Centre
HEMS: Helicopter Emergency Medical Services
HPD: HEMS paramedic dispatcher
IQR: Inter-quartile range
KSS: Kent, Surrey & Sussex
KSSAAT: Kent, Surrey & Sussex Air Ambulance Trust
NCD: Non-clinical dispatcher
OR: Odds Ratio
